# Survive or thrive: tradeoff strategy for cellular senescence

**DOI:** 10.1038/emm.2017.94

**Published:** 2017-06-02

**Authors:** Sang Chul Park

**Affiliations:** 1 Well Aging Research Center, Department of New Biology, DGIST, Daegu, Korea

## Abstract

Aging-dependent cellular behaviors toward extrinsic stress are characterized by the confined localization of certain molecules to either nuclear or perinuclear regions. Although most growth factors can activate downstream signaling in aging cells, they do not in fact have any impact on the cells because the signals cannot reach their genetic targets in the nucleus. For the same reason, varying apoptotic stress factors cannot stimulate the apoptotic pathway in senescent cells. Thus, the operation of a functional nuclear barrier in an aging-dependent manner has been investigated. To elucidate the mechanism for this process, the housekeeping transcription factor Sp1 was identified as a general regulator of nucleocytoplasmic trafficking (NCT) genes, including various nucleoporins, importins, exportins and Ran GTPase cycle-related genes. Interestingly, the posttranslational modification of Sp1 is readily influenced by extrinsic stress, including oxidative and metabolic stress. The decrease in SP1 *O*-GlcNAcylation under oxidative stress or during replicative senescence makes it susceptible to proteosomal degradation, resulting in defective NCT functions and leading to nuclear barrier formation. The operation of the nuclear barrier in aging provides a fundamental mechanism for cellular protection against stress and promotes survival at the expense of growth via stress-sensitive transcriptional control.

## Introduction

Among the several characteristic features of senescent cells, including morphologic enlargement, senescence-associated β-galactosidase activity, reduced response to growth factors, increased apoptotic resistance and changes in gene expression, altered cellular responsiveness might be the most effective way to understand the phenotypic behaviors of senescent cells.^1, 2, 3, 4^ Cellular responsiveness can be classified into three different categories. The first category is the metabolic response to nutrient supply and utilization. The metabolic response should be maintained intact for homeostasis regardless of age. The second category is the stress response against a variety of toxic stress. All cells and organisms are confronted with toxic or apoptotic stress. However, it is generally accepted that stress responses are attenuated with the age of the cell or organism. The third category is the mitogenic response. In aging, it is well known that responses to growth factors are distinctively reduced or even blocked. Responsiveness to stress and mitogenic factors is more attenuated or damaged than the metabolic response in aging cells and organisms.^3^ Therefore, it might be intriguing to elucidate the mechanism underlying these aging-dependent changes in cellular responsiveness.

## Reduced responsiveness to growth factors: Barrier to thrive

To determine cellular responsiveness, many aspects of its signaling system, such as ligand and receptor quantity, ligand-receptor affinity, downstream signaling cascades, intracellular networks, and signal translocation, transcriptional control, chromatin remodeling, and posttranscriptional status, should be assessed.^4^ Growth factors stimulate their respective receptors and subsequently activate downstream signals. However, senescent cells, in general, have a reduced efficiency of response to external growth factors.^5, 6, 7^ Senescent cells exhibit downregulation of mitogenic response toward EGF, despite questionable changes in the amounts of ligands or receptors.^2, 4, 6, 8^ However, it has recently been shown that the functional recovery of senescent cells, especially with regard to the mitogenic response, could be induced to some extent simply through the adjustment of clathrin-dependent or -independent receptor-mediated endocytosis.^9^

The major component of caveolae is caveolin-1, which is abundant in terminally differentiated cell types and which is located at the sites where signaling molecules are concentrated.^10, 11, 12, 13^ Caveolae, the subcellular compartments for storing and regulating signaling molecules, facilitate crosstalk between signaling cascades. The interaction of caveolin-1 with signaling molecules, such as G protein alpha-subunits, H-Ras, Src-family tyrosine kinases, PKC isoforms, EGFR, Neu and eNOS, is mediated via its membrane-proximal region, which is called the caveolin-scaffolding domain.^14, 15, 16, 17^ The targeted downregulation of caveolin-1 is sufficient to drive the transformation of cells and to activate the Erk kinase cascade. Co-expression of EGFR with caveolin-1 results in the suppression of signal transduction from the cytoplasm to the nucleus *in vivo*.^18^ Senescent human diploid fibroblasts (HDFs) have an elevated level of caveolin-1, which colocalized with EGFR.^5^ Overexpression of caveolin-1 reduced the activation of Erk-1/2 after EGF stimulation, suggesting a direct role for caveolin-1 in EGF signaling, followed by the induction of premature cellular senescence of murine fibroblasts.^4, 19^ By contrast, downregulation of caveolin-1 led to the restoration of basal p-Erk levels and Erk activation in response to EGF stimulation with the downstream activation of Elk phosphorylation in senescent HDFs. The activation of Erk signaling by its phosphorylation through PP1 and PP2A is also affected by caveolin-1.^20^ Cell cycle arrest by caveolin-1 is controlled by the p53/p21Waf1-dependent pathway.^21^ These results suggest the possibility of modulating the aging phenotype by the adjustment of the level of caveolin-1 in senescent cells.^5^

Furthermore, caveolin-1 is linked to the focal adhesion complex via integrins in the membrane, which implicates it in the control of focal adhesion and the adhesion signaling cascade.^22, 23, 24^ The formation of focal adhesion and actin stress fibers is relatively higher in senescent cells, and they are anchored to the membrane via interaction with caveolin-1. Therefore, the process of restoring the shape of senescent cells in order for them to resemble young cells by adjusting focal adhesion complexes via the control of caveolin-1 could be activated.^5, 25, 26^

The restoration of the senescent phenotype to a functionally active and young state by adjusting the caveolin-1 status illustrates the significant value of the membrane signaling system in aging.^27^ Therefore, it has been speculated that the aging process can be initiated and modulated at the membrane by a membrane-associated signal switch system. These data led to the emergence of the gate theory of aging, in which the fundamental role of a membrane switch system has been emphasized.^5, 28^ The gate theory of aging strongly suggests the possibility of restoration of the young phenotype in senescent cells by modulating the signaling system on the cell membrane. This novel hypothesis changes the conventional idea of aging as an irreversible and inevitable process into that of a flexible and plastic process.^3, 29^

## Enhanced resistance to apoptotic stress: Strategy to survive

Resistance to age-dependent apoptosis has been reported both *in vitro* and *in vivo*.^30, 31^ Apoptosis in many physiological and pathological processes of aging and of age-related diseases is intimately connected to MAPKs and the serine/threonine kinases that phosphorylate specific substrates, respectively. ERK1 and ERK2 are well-characterized MAPKs that are activated mainly in response to growth stimuli, while JNKs and p38-MAPK are activated in response to a variety of stressors, including DNA damage, heat shock, ischemia, inflammatory cytokines, UV radiation and oxidative stress.^32, 33, 34, 35, 36, 37^ The phosphorylation of MAPKs can be controlled in an agonist-specific manner.^38, 39, 40^ Hydrogen peroxide, an inducer of apoptosis, promotes an increase in ERK phosphorylation.^41^ Staurosporine, a strong inducer of caspase 3-dependent apoptosis, activates p38 phosphorylation.^42^ However, most of the signaling molecules can execute their respective functions only after their entry into the nucleus. Thus, it can be speculated that apoptotic resistance in senescent cells might be closely linked to defects of the apoptotic signals that prevent entry into nuclei.^3^

In mammalian cells, the ratio of proapoptotic proteins and anti-apoptotic Bcl-2 family members is important in determining whether and when apoptosis is triggered.^43, 44^ The levels of Bcl-xL and Bax were comparable in young and senescent HDFs, but Bak, Bok, Bik, and PUMA were present at lower levels in senescent HDFs than in young HDFs.^3, 40^ These data imply that the lower level of expression of the proapoptotic genes might be related to the failure of the signaling molecules to trigger their expression, thereby desensitizing the senescent cells to apoptotic stimuli. By contrast, the stable expression of anti-apoptotic Bcl-2 in senescent cells despite apoptotic stress might be related to the phosphorylation status of CREB due to the inactivation of protein phophatase-2A (PP2A), as well as the inhibition of the apoptotic signal transduction into the nucleus.^40^ Moreover, the senescence-dependent nuclear accumulation of actin, gelsolin^45, 46^ and major vault protein (MVP)^38^ strongly support the specific localizing mechanism of the signal to either the cytosol or the nucleus. Therefore, we hypothesized that senescence-dependent defects in intracellular signaling, especially in nucleocytoplasmic trafficking, might provide a mechanism for cellular resistance to extrinsic stress.^3^

## Defective nucleocytoplasmic trafficking: From flexibility to fixation

Many signaling molecules and transcription factors need to enter the nucleus to exert their respective effects. For example, EGF stimulation induces the entry of p-ERK into the nucleus, which results in the activation of the transcription factor AP1, triggering cell cycle progression.^5, 47^ The apoptotic response is a well-programmed process that also requires the entry of intracellular signaling molecules into the nucleus. Therefore, it is natural to assume that the lack of responsiveness to both apoptotic stress and growth factors in senescence is related to the inefficiency of nucleocytoplasmic trafficking in the senescent cells. Nucleocytoplasmic trafficking is a highly sophisticated process that involves many component proteins. Nucleoporins are the major components of nuclear pore complexes (NPCs), often used as markers for NPCs.^48, 49^ NPCs allow the passive diffusion of ions and small molecules, and facilitate the active transport of macromolecules. The cargo molecules usually have short sequence elements called nuclear localization sequences and nuclear export sequences. Karyopherin α binds to the nuclear localization sequences of cargo molecules, while karyopherin β binds to both karyopherin α and nucleoporins.^50, 51^ Ran plays an important role in the import and export of cargoes, and is present in two distinct forms: GTP-bound Ran and GDP-bound Ran. The nucleotide state of Ran is regulated by Ran GTP-GDP exchange factor (RanGEF or RCC1) and Ran GTPase-activating protein (RanGAP).^52, 53^

The initial observation of aging-dependent nuclear accumulation of actin^46^ and gelsolin,^45^ as well as the perinuclear accumulation of many signaling molecules in response to a variety of apoptotic and mitotic stress conditions,^39, 40, 54, 55^ implied that the nuclear translocation of activated signaling molecules might be inhibited by a certain barrier at the nuclear membrane, as shown by the perinuclear confinement of p-ERK1/2 and the p50 subunit of NF-kB in response to growth stimuli or LPS in senescent fibroblasts.^3, 55^ However, the activation of these signaling molecules is apparently not impaired, as illustrated by the efficient phosphorylation and activation of ERK1/2 via its interaction with PKCα or PP1 and PP2A in senescent cells.^56, 57, 58^ These findings assert that the signaling molecules cannot be distributed properly to the nucleus by simple activation but that they need the proper operation of the nucleocytoplasmic trafficking system under senescent conditions.

This nucleocytoplasmic trafficking system operates physiologically in response to external stimuli. In young and healthy cells, this system is readily influenced by either growth stimuli or nutritional condition. When the cellular energy state is low, the AMPK pathway is activated, resulting in the import of biomolecules into the nucleus. By contrast, when the cell receives growth stimuli, the PI3K signaling cascade is activated, leading to the export of biomolecules out of the nucleus, as demonstrated by GAPDH (glyceraldehyde 3-phosphate dehydrogenase).^59^ These data illustrate the plastic nature of nuclear translocation in cellular homeostasis. However, the operation of this trafficking system is strictly controlled in the senescent state.^3^

Many studies have been carried out on the structural and functional aspects of the nuclear trafficking system, as it is one of the essential features of metazoan life.^60^ Concerning the study of nuclear trafficking in aging, though the nature of its suppression and the reduction of NPC components have been acknowledged, the underlying mechanism for its loss of function remains unclear and disputed.^61^ The oxidative stress-dependent loss of nuclear trafficking by the oxidation of NPC components has previously been illustrated.^62, 63, 64^ Furthermore, aging-dependent leakage of the nuclear membrane has been suggested for the functional deterioration of nuclear trafficking.^65, 66^ However, these reports are still unable to explain the aging-dependent dynamics of the nuclear trafficking system and the mechanistic regulation of functional hyporesponsiveness of senescent cells toward both growth and death-inducing stress.

## Dynamics of nucleocytoplasmic trafficking: Operation of the nuclear barrier

Microarray analysis revealed that most of the nucleocytoplasmic trafficking genes, including most nucleoporin and transport receptor genes, as well as Ran and Ran-regulating factors, were downregulated in senescent HDFs.^55^ To verify the microarray data, the expression levels of some of the selected genes were confirmed with a semi-quantitative RT-PCR method and the protein levels were validated by western blotting with available antibodies.^54, 55^ These results showing the suppression of the nucleocytoplasmic trafficking genes and proteins in senescent HDFs strongly support the presumed defective operation of the nucleocytoplasmic trafficking system.^3^

The senescence-dependent reduction of nucleocytoplasmic trafficking gene expression was traced to its ultimate regulator through analysis of the upstream transcription factors for those genes. With bioinformatics tools, all the nucleocytoplasmic trafficking genes were subjected to promoter analysis for putative transcription factors. Among these transcription factors, Sp1 (specificity protein 1) was selected as the most common and dominant transcription factor responsible for the genetic control of the trafficking-associated genes. Furthermore, it was confirmed that most of the promoters of the trafficking-associated genes had multiple Sp1 binding sites.^3, 55^

However, Sp1 stability was found to be damaged in senescent HDFs, and the DNA-binding activity of Sp1 was reduced in aged brain and liver tissues.^67, 68^ Sp1 protein levels were lower in senescent cells compared with young cells and in various tissues of aged mice compared with those from young mice. These results additionally support the possibility of Sp1 as a good candidate for the master regulation of NCT genes, showing that the reduced Sp1 protein levels in senescent cells and tissues could be a common causal factor for aging-dependent suppression of the NCT genes.^55, 69^

To test whether Sp1 was a common regulator for the expression of NCT genes, changes in NCT gene expression were determined after the modulation of Sp1 expression. Knockdown of Sp1 by si-Sp1 in young HDFs globally downregulated NCT genes. However, some nucleoporins, transport receptors and Ran GTPase cycle-related genes were heavily downregulated by Sp1 depletion, and the protein levels of Nup50, Nup88, Nup107, Nup155, karyopherin α2 and RCC1 were decreased in the Sp1-depleted HDFs.^55^ By contrast, Sp1 overexpression induced a general up-regulation of the NCT genes, and the protein levels of Nup50, Nup88, Nup107, Nup155, karyopherin α2 and RCC1 were also shown to be up-regulated. These results support the hypothesis that Sp1 is a common, functionally active master regulator of NCT gene expression.^69^ Additionally, the direct interaction of Sp1 with the promoter regions of NCT genes was confirmed by ChIP (chromatin immunoprecipitation) analysis (Ryu *et al.*, submitted).

In order to determine the functional role of Sp1, the nuclear translocation of p-ERK1/2 was tested in response to EGF stimulation in either Sp1-depleted or Sp1-overexpressing cells. The majority of p-ERK1/2 molecules were present in the nuclear fraction after EGF stimulation in young HDFs, while this nuclear localization was significantly attenuated in the si-Sp1-transfected cells. On the other hand, the majority of p-ERK1/2 was detected in the cytoplasmic fraction of senescent cells, and Sp1 overexpression significantly facilitated their nuclear localization.^69^ Furthermore, to confirm the role of Sp1 in the modulation of nuclear translocation of the signaling molecules, its downstream signaling was tested. Thus, the phosphorylation status of Elk, a known substrate of ERK1/2, and the expression of c-fos mRNA, a p-Elk target gene, were monitored. Elk phosphorylation at Serine 383 was markedly decreased in the Sp1-depleted cells compared with that in the young control cells, and was accompanied by a significant reduction of c-fos mRNA. These results strongly suggest that Sp1 influences the nucleocytoplasmic trafficking of signaling molecules, as well as their downstream signaling by modulating the efficiency of NCT gene expression. These data also strengthen the assumption that reduced Sp1 protein levels might be a causal factor for aging-dependent hyporesponsiveness to extrinsic stress.^69^

## Maintenance of the nuclear barrier in aging: requirement for continuing stress

In order to identify the mechanism of downregulation of the Sp1 protein level under senescent conditions, the Sp1 mRNA level was determined, but no significant difference between young HDFs and senescent HDFs was obtained, implying the posttranslational regulation of Sp1 protein rather than its transcriptional control. As reactive oxygen species (ROS) levels are high in senescent HDFs,^70, 71^ Sp1 protein levels in response to H_2_O_2_ treatment were tested. The data demonstrated that the Sp1 protein level was strikingly reduced by H_2_O_2_ treatment, which was readily blocked by *N*-acetylcysteine (NAC), a radical scavenger, supporting the hypothesis that the aging-related increase in ROS could be responsible for Sp1 protein downregulation. In addition, to determine whether the ROS-induced modification of the Sp1 protein was relevant for its proteasome-mediated degradation, Sp1 protein levels were determined with or without the proteasome inhibitor N–Ac–Leu–Leu-norleucinal (ALLN). The H_2_O_2_-dependent reduction of the Sp1 protein level was prevented by ALLN in both young and senescent HDFs, suggesting that Sp1 protein level is downregulated by ROS via proteasome-mediated degradation during the aging process.^69^

**Figure 1:**
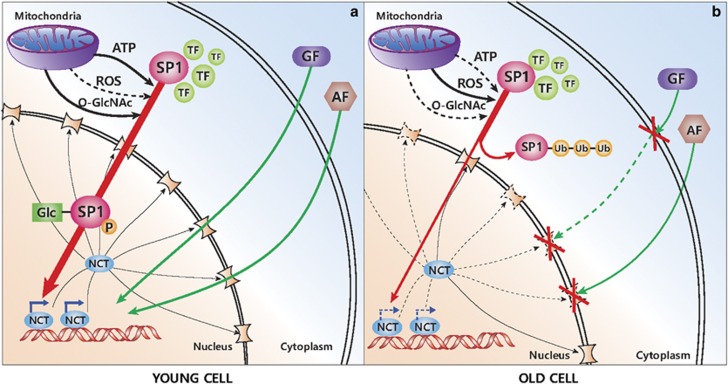
Comparison of nucleocytoplasmic trafficking between young and old cells. (**a**) Young cell. Sp1 can activate the transcription of NCT genes, and the nuclear pores are functioning well for both mitogenic and apoptotic signals. (**b**) Old cell. Sp1 is vulnerable to degradation, resulting in defective functioning of nuclear pores, which restricts signal trafficking. GF, growth factor; AF, apoptosis-inducing factor; TF, transcription factor; NCT, nucleocytoplasmic trafficking genes; *O*-GlcNAc, *O*-linked N-acetyl glucosamine; ub, ubiquitination; thick arrow, high activity; thin arrow, low activity; dotted line, restricted activity.

Because *O*-GlcNAcylation has been suggested as a mechanism for Sp1 stability,^72^ the *O*-GlcNAcylation status of Sp1 was compared between young and senescent HDFs. A marked decrease in the *O*-GlcNAcylation status of Sp1 was seen in senescent cells when compared with young cells. Furthermore, the level of *O*-GlcNAcylated Sp1 was significantly decreased by H_2_O_2_ treatment in the presence of ALLN.^69^ These data suggest that ROS can reduce the *O*-GlcNAcylation of Sp1, and can consequently facilitate its proteasome-mediated degradation in aging. In other words, the aging-related increase in ROS aggravates the hypo-*O*-GlcNAcylation of Sp1, followed by its consequent degradation. The dysfunction in glucose metabolism in senescent cells^73^ likely further limits the *O*-GlcNAcylation of Sp1 to a low level in general by reducing hexosamine turnover, which requires high metabolic energy.^69^ This mechanism of aging-dependent nuclear barrier formation is summarized in Figure 1.

Sp1 is a transcription factor that has long been regarded as a regulator of housekeeping genes, and it has been well characterized for its structure and functions. Notably, its knockout in mice causes embryonic lethality with a broad range of phenotypic abnormalities, implicating a versatile function in many cell types.^74, 75, 76^ Chromosomal mapping studies of the human genome elucidated the presence of at least 12 000 Sp1 binding sites.^77^ Sp1 is active in all cell types, and its activity is tightly regulated in response to signaling pathways and changing cellular conditions, which further affects its interaction with a variety of binding partners to regulate Sp1-dependent transcription.^78, 79^ Considering the role of Sp1 in a multitude of cellular pathways and processes, it is natural to assume its association with the pathogenesis of a number of diseases, especially cancer. Sp1 overexpression has been observed in many cancer cell types, where the levels of Sp1 correlate with tumor stage,^76^ leading to the development of anticancer agents that inhibit the action of Sp1.^80^

However, the conflicting bifunctional behavior of Sp1 in cell growth and other biological phenomena limits our understanding of its genuine biological role. For example, Sp1 regulates the genes responsible for the progression of the cell cycle and entry into S-phase, such as cyclins and *MYC*, as well as growth factor signaling pathways, such as *IGF1R*.^81, 82^ Sp1 also controls the transcription of cell cycle inhibitor genes, such as *p21*, synergizing with p53 under conditions of cellular stress.^83, 84^ Moreover, the expression of telomerase subunits required for the maintenance of telomeres and cell immortality is also controlled by Sp1 through the Sp1 binding sites at the *hTERT* promoter,^85^ while the interaction of SP1 with HDACs represses *hTERT* expression.^86^ Although only a few examples are illustrated here, Sp1 and its family members have a broad spectrum of biological functions operating in both pro- and anti-cell growth, which makes it difficult to discern their genuine biological functions. However, the novel concept of the role of Sp1 in the regulation of NCT function provides a new breakthrough in our understanding of its biological functions, as NCT is essential and strongly associated with most biological phenomena, including growth, differentiation, metabolism, apoptosis, cancer and senescence. Moreover, the dynamic nature of the Sp1 protein due to posttranslational modifications, such as phosphorylation, glycosylation, acetylation, poly (ADP-ribosyl)ation, methylation, sumoylation and oxidation,^87, 88, 89, 90, 91^ makes it sensitive to extrinsic stress, thereby implying its value as a regulator of biological responsiveness.

In addition, it is evident that many other transcription factors other than Sp1, as well as many NPC components, are also vulnerable to oxidative stress, leading to their loss of function.^62, 63, 64^ However, it can be assumed that the overall control of regulation of NPC formation by Sp1 is more powerful and effective for the aging process than the other individual components combined.

Taken together, it can be speculated that because Sp1 is one of the housekeeping genes, a continuous input of stress signals, whether metabolic or oxidative, would be required to maintain the reduced level of Sp1 protein, finally leading to senescence. All these data support the hypothesis that a functional nuclear barrier of hyporesponsiveness, called the ‘Park and Lim’s Barrier’, to external stimuli is operating in senescent cells, in relation to the downregulation of the common transcription factor, Sp1.^3^ This Sp1 status-dependent nuclear barrier provides the mechanism of aging as a tradeoff between growth arrest and apoptosis resistance. The vulnerable nature of Sp1 protein status that is regulated by a variety of posttranslational modifications in response to nutritional supply and varying stress further supports the role of Sp1 as one of the prime determinants of the senescent phenotype. In addition, it can be speculated that, through the adjustment of Sp1 status, the nucleocytoplasmic trafficking defects of senescent cells can be restored and the aging phenotype can be reversed. It has already been shown that Sp1 overexpression can induce the restoration of the telomere damage-dependent senescent phenotype to a young phenotype to some extent.^92^ Therefore, there is no doubt that the modulation of this barrier mechanism simply via changing Sp1 status may open a new avenue for the adjustment of nuclear trafficking in senescence, cancers and neurodegenerative disorders.

## Conclusion

The features of hypo-responsiveness of senescent cells either to growth factors or to apoptotic stress are related to a functional nuclear barrier with defective nucleocytoplasmic trafficking resulting from the transcriptional downregulation of the relevant genes. This barrier is important for the survival of the aging cells at the expense of growth and provides a platform for regulating the crosstalk between good and bad signals from extrinsic sources. In addition, the transcription factor Sp1 has been implicated in the regulation of this barrier, and, through simple adjustments of Sp1 levels, the nucleocytoplasmic trafficking defects of senescent cells can be restored, and consequently, the aging phenotype can be reversed. Furthermore, it can be assumed that this mechanism may provide new insights for novel therapeutic modalities in cancers, neurodegenerative disorders and other aging-related diseases by adjusting the nucleocytoplasmic trafficking status.
